# An observational feasibility study - does early limb ergometry affect oxygen delivery and uptake in intubated critically ill patients – a comparison of two assessment methods

**DOI:** 10.1186/s12871-020-01227-z

**Published:** 2021-01-25

**Authors:** Olive M Wilkinson, Andrew Bates, Rebecca Cusack

**Affiliations:** 1 Centre for Innovation and Leadership, Faculty of Health Sciences, University of Southampton, Building 45, Room 2035, Highfield Campus, S017 1BJ Southampton, UK; 2 Critical Care Anaesthesia and Perioperative Research Unit and Integrative Physiology, Clinical Experimental Sciences and NIHR Respiratory Biomedical Research Unit, University Hospital Southampton NHS Foundation Trust and University Hospital Southampton, Southampton, UK

**Keywords:** Early rehabilitation, Physiological response, Critical care

## Abstract

**Background:**

Early rehabilitation can reduce ventilation duration and improve functional outcomes in critically ill patients. Upper limb strength is associated with ventilator weaning. Passive muscle loading may preserve muscle fibre function, help recover peripheral muscle strength and improve longer term, post-hospital discharge function capacity. The physiological effects of initiating rehabilitation soon after physiological stabilisation of these patients can be concerning for clinicians. This study investigated the feasibility of measuring metabolic demand and the safety and feasibility of early upper limb passive ergometry. An additional comparison of results, achieved from simultaneous application of the methods, is reported.

**Methods:**

This was an observational feasibility study undertaken in an acute teaching hospital’s General Intensive Care Unit in the United Kingdom. Twelve haemodynamically stable, mechanically ventilated patients underwent 30 minutes of arm ergometry. Cardiovascular and respiratory parameters were monitored. A Friedman test identified changes in physiological parameters. A metabolic cart was attached to the ventilator to measure oxygen uptake. Oxygen uptake was concurrently calculated by the reverse Fick method, utilising cardiac output from the LiDCO™ and paired mixed venous and arterial samples. A comparison of the two methods was made. Data collection began 10 minutes before ergometry and continued to recovery. Paired mixed venous and arterial samples were taken every 10 minutes.

**Results:**

Twelve patients were studied; 9 male, median age 55 years, range (27–82), median APACHE score 18.5, range (7–31), median fraction inspired oxygen 42.5%, range (28–60). Eight patients were receiving noradrenaline. Mean dose was 0.07 mcg/kg/min, range (0.01–0.15). Early ergometry was well tolerated. There were no clinically significant changes in respiratory, haemodynamic or metabolic variables pre ergometry to end recovery. There was no significant difference between the two methods of calculating VO_2_ (*p* = 0.70).

**Conclusions:**

We report the feasibility of using the reverse Fick method and indirect calorimetry to measure metabolic demand during early physical rehabilitation of critically ill patients. More research is needed to ascertain the most reliable method. Minimal change in metabolic demand supports the safety and feasibility of upper limb ergometry. These results will inform future study designs for further research into exercise response in critically ill patients.

**Trial Registration:**

Clinicaltrials.gov No. NCT04383171. Registered on 06 May 2020 - Retrospectively registered. http://www.clinicaltrials.gov.

## Background

In 2017–2018 there were over 290,000 adult Intensive Care Unit (ICU) patient records in England, of whom 7.4% had a length of stay duration of eleven days and more [1] In the critically ill patient muscle wasting can occur within hours of the initiation of mechanical ventilation and may be exacerbated by multi-organ failure, heavy sedation and immobility [2–4]. The consequences for survivors include long-term physical disability, poor quality of life and increased associated health care costs [5, 6].

A number of studies have reported that the early application of ICU mobility therapy can reduce the number of ventilator days, ICU and hospital length of stay [7, 8] as well as improving functional outcome at hospital discharge [9, 10]. Although, early ICU mobility therapy studies have reported minimal safety issues, there are concerns that such activities, undertaken during the acute phase of critical illness may result in additional, unreported metabolic demands.

Oxygen consumption is a widely used proxy measure for metabolic rate. Within the ICU, whole body oxygen consumption can be determined by using paired arterial and venous blood oxygen content, in conjunction with cardiac output monitoring. (The reverse Fick method). Alternatively, indirect calorimetry techniques provide a minimally invasive assessment of energy consumption, using a metabolic cart, attached to the ventilator measures breath by breath, inhaled and exhaled oxygen and carbon dioxide. [11]. However, the precision and reliability of indirect calorimetry in the critically ill patient has been questioned [12].

The primary objective of our study was to assess the feasibility of using the reverse Fick method and indirect calorimetry to measure metabolic demand and to assess the safety and feasibility of passive upper limb cycle ergometry, during the first days of critical illness. A secondary objective analysis will compare simultaneous assessment of the two methods of assessing oxygen uptake (VO_2_). We hypothesize that measuring oxygen consumption during passive exercise in critically ill patients is feasible and will demonstrate minimal metabolic demands on the patient. This study is reported in accordance with the STROBE statement [13].

## Methods

This observational feasibility study was conducted in a 25-bed University Teaching Hospital ICU in the United Kingdom. Ethical approval was granted by South Central – Hampshire National Research and Ethics Service. Reference: 14/SC/1398.

### Patient recruitment

All patients admitted to the ICU with a medical diagnosis and requiring intubation and ventilation for at least 48 hours join an Early Mobility Program (EMP). This programme provides a progressive mobility pathway starting with daily passive upper/lower limb exercise sessions by means of an ergometer in addition to their routine physiotherapy. Inclusion criteria for enrolment into the study included: patient being on the EMP pathway of which the criteria were cardiovascular stability (stable vasopressor dose for two hours); stable heart rate (< 140 bpm) and rhythm and the presence of a jugular central venous pressure (CVP) line and arterial line. Exclusion criteria included any prior rapidly deteriorating neuromuscular disease, any upper limb problem precluding cycle ergometry, pyrexia (temp > 38 °C), raised intracranial pressure, patients with poor prognostic outcomes and lack of agreement from clinician or NOK/LR not understanding English. 149 patients accepted onto the EMP were screened for study eligibility.

All included patients had monitoring in place that included: electrocardiogram (ECG) set, saturation probe, CVP line and an arterial line. Patients were sedated according to the ICU sedation protocol using a combination of fentanyl, midazolam and/or propofol aiming for a RASS score between − 1 and + 1. Vasopressors were used to maintain a mean arterial blood pressure of > 75 mmHg. Sedative infusion rates and ventilation settings were not changed during the protocol.

Patients were ventilated using pressure or volume control modes or support mode using an Engstrom Carestation™ ventilator. Flow volumes were directly measured by a ventilator D-lite™ sensor, which measures pressure difference between two ports and calculates gas/air flow (GE Healthcare, Chicago, Illinois).

### Early mobility interventions

Patients were positioned in bed, in a semi recumbent position with their arms in the limb supports of the cycle ergometer (MOTOmed letto2 – Reck, Reckstr 1–5, Betzenweiler 88,422, Germany). Sixty minutes of data was collected. The first 10 minutes were with patients upper limbs positioned in the limb supports and at rest followed by 30 minutes passive upper limb cycling at a frequency of 20 revolutions per minute, and finally 20 minutes with the patients upper limbs left in the limb supports during which the patient was at rest undisturbed. Safety criteria used for patient initiation and continued use of the ergometer was based on the traffic light system recommended from Hodgson et al. [14] .

### Measurements

Continuous heart rate (HR), blood pressure (BP), heart rhythm and saturations were measured throughout the 60-minute study period for each patient. Continuous cardiac output (CO) L/min, HR (bpm), BP (mmHg), and stroke volume (SV) m/L were monitored by the LiDCO™. The LiDCO™ was calibrated as per manufacturer guidance, prior to patient enrolment. Minute by minute values of inspiratory and expiratory O_2_ and CO_2_, respiratory rate (RR) breaths/min, minute volume (MV) mL and tidal volume (V_T_) mL were measured by the ventilator's E-COVX module. Values for all continuous data were averaged over the five minutes leading up to each 10-minute interval within the 60-minute study period. If any data was missing within those last five minutes the average was calculated by the number of available data within that time frame.

Paired central mixed venous blood and arterial blood gas samples were taken at 10 minutes prior to ergometry starting, and then at 0, 10, 20 and 30 minutes, during ergometry and again 10 and 20 minutes after ergometry finished.

Oxygen delivery (DO_2_) was calculated using the equation: DO_2_ = CaO_2_ x CO where CaO_2_ (arterial oxygen content) = (1.34 x Hb x SaO_2_ x 0.01) + (0.023 x PaO_2_). The value 1.34 is known as Hufners constant and 0.023 is the volume of O_2_ dissolved per 100 ml plasma per kPa.

CO_2_ production (VCO_2_) was calculated from values of inspired concentrations of CO_2_ (FiCO_2_) and expired concentrations of CO_2_ (FeCO_2_) by the E-COVX module via the ventilator using the Bohr equation: VCO_2_ kPa = FiCO_2_ – FeCO_2_. Oxygen uptake (VO_2_) was calculated by two methods.

1. Method one: The reverse Fick method uses the measure of CO from the LiDCO™ with paired central mixed venous and arterial blood gas samples: VO_2_ mL/min = CO x (CaO_2_ - CvO_2_) x10 [15].
2. Method two: Indirect calorimetry calculated VO_2_ using the E-COVX metabolic module via the ventilator from the value of fraction of inspired O_2_ (FiO_2_), expiratory minute volume (MV), expired concentrations of O_2_ (FeO_2_) and CO_2_ (FeCO_2_) using the equation: VO_2_ ml/min = MV (FiO_2_ –FeO_2_ – FiO_2_ (FeCO2))/1-FiO_2_. [16]

### Statistical analysis

This is a feasibility study the results of which may be used to power a larger study if appropriate. The study population number was guided by previous work in our unit [17]. All statistical analyses were performed using the SPSS 11 for Mac OS X (version 11.0.2). Demographics were presented for each patient. Continuous data was presented graphically as the mean for the five minutes leading up to each blood gas sampling, unless otherwise stated, except for the first blood sample. For repeated measures an analysis of variance was carried out using the Friedman test to determine any changes occurring in the physiological parameters from baseline to the six different time points. Correlation between the methods was assessed using a Pearson’s correlation. The significance difference was set at p < 0.05. Percentage changes in physiological parameters are expressed as interquartile range (IQR) and range.

## Results

### Feasibility

**Fig. 1:**
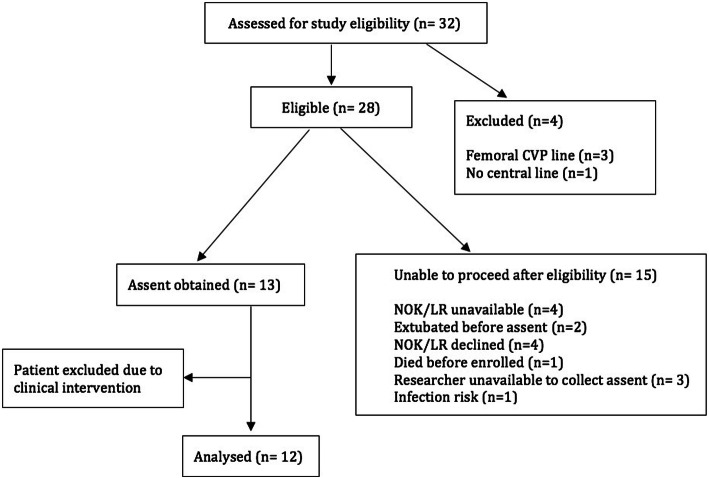
Flow of participants

From the convenience sample of 32 patients were assessed for eligibility, four patients did not meet the inclusion criteria thirteen patients gave assent and twelve patients were studied (Fig. 1). All patients were intubated and ventilated, two by a mandatory mode, nine by assist mode and one on continuous positive airway pressure (CPAP). Mean positive end expiratory pressure (PEEP) was 9 cm H_2_O with a range of 0 to 12 cm H_2_O (Table 1).

**Table 1:** Demographic and clinical data of patient’s pre ergometry session

Patient no.	0001	0002	0003	0004	0005	0006	0007	0008	0009	0010	0011	0012
**Age - yrs.**	60–69	50–59	60–69	40–49	50–59	30–39	70–79	30–39	50–59	80–89	30–39	20–29
**BMI**	24.9	21.4	21	27	27.1	22.9	24.2	47.2	17.3	32.4	23.3	27
**APACHE II score**	23	16	19	19	18	7	31	20	18	26	10	13
**RASS**	-4	-5	-3	+ 1	-3	-4	-3	-4	-4	-4	+ 1	-4
**Ventilator mode (start of ergometry session)**	Volume cycled/ PEEP 14 cm H_2_0	Volume cycled/ PEEP 12 cm H_2_0	PS/ PEEP 14/10 cm H_2_0	PS/ PEEP 10/12 cm H_2_0	CPAP 5 cm H_2_0	PS/ PEEP 10/5 cm H_2_0	PS/ PEEP 14/5 cm H_2_0	PS/ PEEP 5/12 cm H_2_0	PS/ PEEP 18/10 cm H_2_0	PS/ PEEP 6/12 cm H_2_0	PS/ PEEP 5/14 cm H_2_0	PS/ PEEP 16/0 cm H_2_0
**Vasopressor**	N/ad	N/ad	N/ad	N/ad	N/ad	N/ad	N/ad	Nil	Nil	Nil	N/ad	Nil
**SOFA score**	9	6	8	5	3	5	8	4	4	9	5	3
**FiO**_**2**_**(%)**	50	45	50	28	35	28	28	60	40	45	45	40
**Diagnosis on admission**	AP	OO HA	Septic shock	Septic shock	OD	OO HA	AKI	AP	P	P	OO HA	P

*M* Male, *F* Female; *BMI* Body Mass Index (weight/height^2^); *RASS* Richmond Agitation Sedation Scale; *PEEP* Positive End Expiratory Pressure, *PS* Pressure Support; *CPAP* Continuous Positive Airway Pressure; *N/ad* Noradrenaline; *SOFA* Sequential Organ Failure Assessment; *FiO*_*2*_Fraction of Inspired Oxygen; *AP* Acquired Pneumonia, *OOHA* Out Of Hospital Arrest, *OD* Overdose, *AKI* Acute Kidney Injury, *P* Pancreatitis.

Ten of the 12 patients completed the 30 minutes ergometry protocol. Insufficient data for analysis was obtained from 2 patients; in one patient this was due to equipment failure and the second patient desaturated during the ergometry session due to heavy secretion load. The ergometry was stopped in order for chest physiotherapy to be delivered. The desaturation was not thought to be related to the ergometry itself. Data was collected for both of these patients up to the point of ergometry cessation. One of the patients who completed the protocol had recurrent muscle contractions during the ergometry session resulting in a 15 second ergometer pause but restarted immediately again and all data was collected and analysed. There are incomplete data sets for VO_2_ calculations for patient No.2, 7 and 12 due to blood sampling difficulties.

### Haemodynamic outcomes

Median arterial blood pressure, systolic and diastolic blood pressure did not change significantly throughout the exercise protocol (*p* = 0.89, *p* = 0.66 and *p* = 0.63 respectively). The median resting HR values did not change throughout the exercise protocol (*p* = 0.60). CO median rest (*n* = 10) value (6.8 L/min, IQR 4.4 L/min) did not change statistically throughout the exercise protocol (*p* = 0.95). There were no significant changes in any of the respiratory variables.

### Metabolic outcomes

**Fig. 2:**
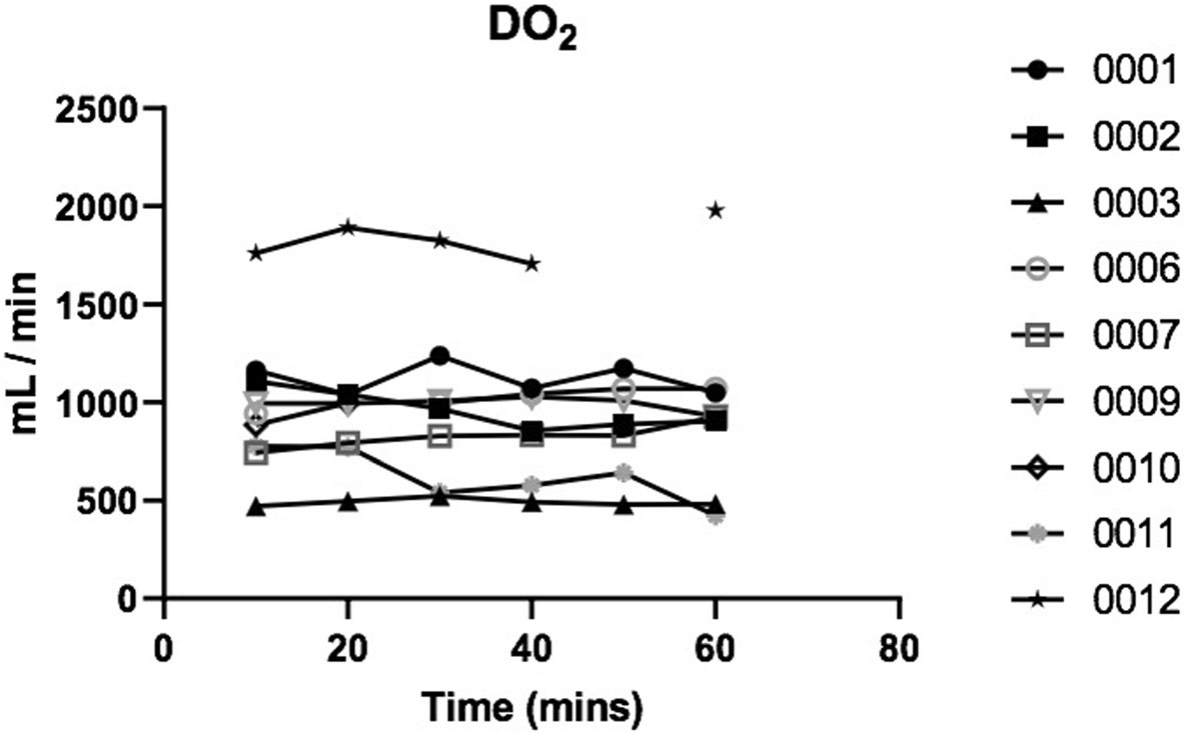
DO_2_ measurements at six different time points during 60-minute data collection period

DO_2_ median rest value was 943 mL/min, (IQR 377 mL/min) (*n* = 9) and did not change statistically from throughout the exercise protocol (*p* = 0.98) (Fig. 2). Maximum change of DO_2_ from baseline was 30.5% for one patient 20 minutes into ergometry session but this reduced to 17.3% at 20 minutes after recovery.

**Fig. 3:**
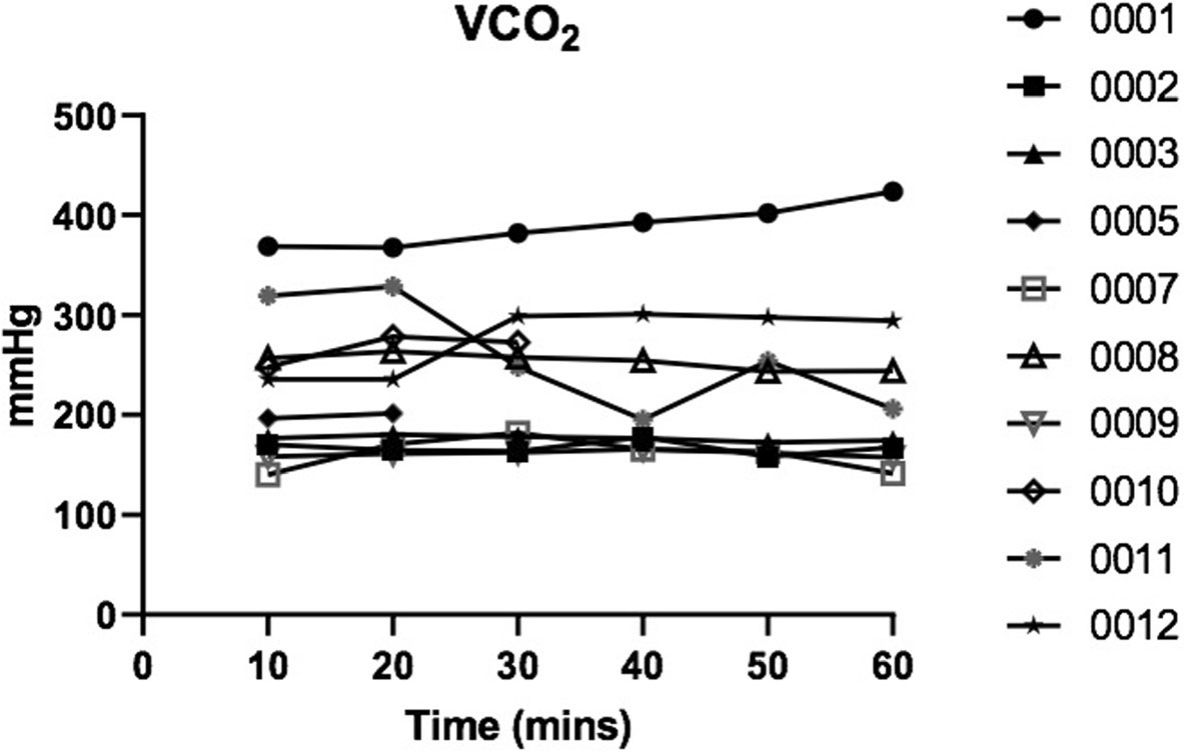
VCO_2_ measurements at six different time points during 60-minute data collection period

ScvO_2_ (n = 12) median rest value was 79.8%, IQR 8.6% and did not change statistically throughout the exercise protocol (p = 0.61). Maximum change of ScvO_2 _during the session was a 15.7% drop during the first ten minutes of ergometry in one patient but this increased to within 1.8% of its resting levels at the end of the exercise session. VCO_2_ (n = 10) median rest value was 216.5 mL/min, IQR 105.5 mL/min and did not change statistically throughout the exercise protocol (p = 0.35). Maximum change of VCO_2_ during the session was a decrease of 38.9% from the median resting value for one patient at the end of the 30-minute exercise session but this increased up to 20.5% from the baseline 10 minutes later (Fig. 3).

### Oxygen uptake (VO_2_)

**Fig. 4:**
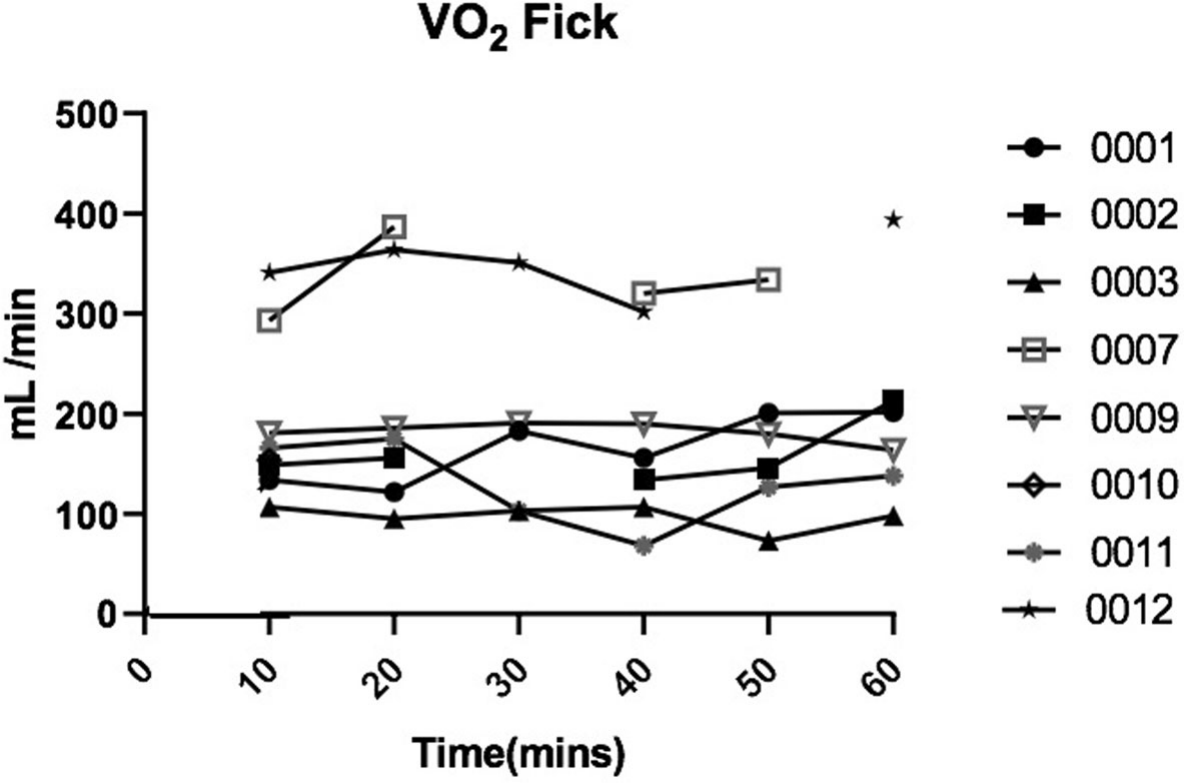
VO_2_ measurements from reverse Fick method at six different time points during the 60 minute data collection period

**Fig. 5:**
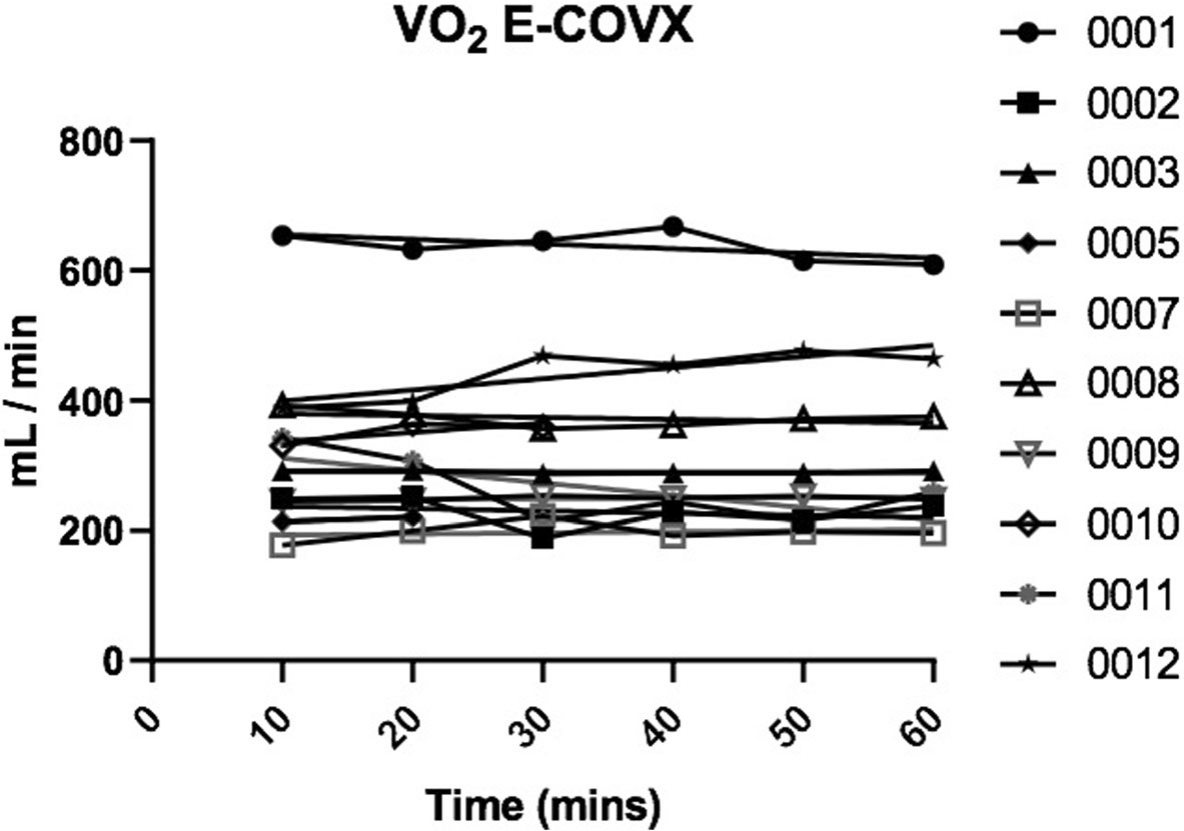
VO2 measurements from E-COVX at six different time points during 60-minute data collection period

Median resting VO_2_ values was 311.5 mL/min, (IQR 152.5 mL/min) and 160 mL/min, (IQR 127.2 mL/min) from the E-COVX (*n* = 10) and the reverse Fick method (*n* = 8) respectively. These did not change statistically throughout the exercise protocol (*p* = 0.95 and *p* = 0.84) (Figs. 4 and 5). The maximum change in VO_2_ from baseline to the end of the 30 minutes exercise was 37.4% using E-COVX and 59.0% using reverse Fick method. The biggest change from baseline post the ergometer stopping, measured by E-COVX, was 24.5% at 20 minutes. The biggest change from baseline post the ergometer stopping, measured by reverse Fick method, was 23.0% at 10 minutes.

**Fig. 6:**
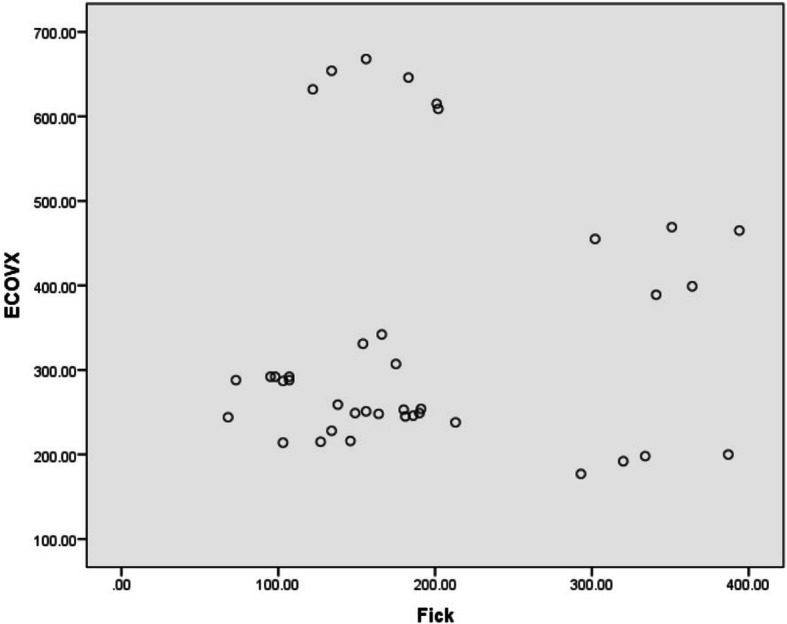
Comparison of both methods of calculating VO_2_ (E-COVX and Fick)

There was poor correlation between the two methods of calculating VO_2_ (r = 0.06) such that bias assessment was not explored (Fig. 6).

## Discussion

This study reports the feasibility of using the reverse Fick method and indirect calorimetry to monitor metabolic response to upper limb ergometry in critically ill patients. Minimal changes in oxygen uptake support the safety and feasibility of early upper limb ergometry. Additional research is required, to determine the most accurate measure of metabolic response.

Desaturation is the most common reported adverse effect seen during early rehabilitation of patients in ICU [18]. There is concern that desaturations may be related to inadequate cardiorespiratory reserve, limiting the capacity to cope with the increased oxygen demand. [19] Our investigation has not supported this finding. The single desaturation event was attributable to heavy secretion load and resolved by respiratory physiotherapy.

Both direct and indirect methods of VO_2_ measurement did not significantly change in response to the cycle ergometry. However, in the majority of patients the VO_2_ measurements using the E-COVX were consistently above those calculated using the reverse Fick method. This warrants further investigation as we cannot account for this discrepancy within the study.

VO_2_ estimation using the reverse Fick method was precluded on a number of occasions due to technical issues with invasive canulae and lines. VCO_2_ data from the E-COVX was very stable throughout our study period. However this method relies on the patient at rest being in a steady state, without this it is susceptible to major errors [16].

A number of studies reporting very early commencement of rehabilitation (within 4 days of ICU admission) have demonstrated improved physical outcomes [8, 20, 21] but there remain concerns that critically ill patients are too ill and unstable to undergo these types of interventions [22].

As muscle breakdown and deconditioning can be demonstrated within hours of mechanical ventilation there is increasing interest on rehabilitation and muscle training within the ICU, however the benefits of these interventions remain uncertain. [3, 23]. It is suggested that mechanical silencing of muscle in the critically ill patients with immobility, sedation, and use of neuromuscular blockade may accentuates muscle breakdown. A number of different methods of passive mechanical loading of muscle in critically ill patients have been used and report reduce wasting, increase muscle strength and being associated with reduced ventilation days and shorter hospital length of stay [23–26]. In bed cycle ergometry is one method of delivering passive muscle loading and on our unit, the early rehabilitation programme has resulted in reduced ventilation days and hospital length of stay [27].

The examination of metabolic costs of the metabolic demands in response to this type of passive mobilization commenced very early during critical illness is not widely studied. A study by Pires-Neto assessing very early passive cycle ergometry, albeit in the lower limbs also found no significant changes in cardio-respiratory or metabolic parameters [28]. In this study the Flotrac-Vigileo (Edwards Lifesciences CA USA) was used to assess cardiac output, however the reliability of the Flotrac has been questioned in dynamic situations.[29].

Few studies have examined VO_2_ in response to other passive interventions although two studies report no significant change in VO_2,_ with either passive chair transfer or physiotherapy treatment [17, 30]. Our study compared two methods of assessing VO_2_. Although the VO_2_ calculations via the E-COVX module in this study produced more variable results than the reverse Fick method, the reproducibility of the reverse Fick method needs to be balanced against the invasiveness of the technique. This difference in results is consistent with previously reported poor correlation and reproducibility between different techniques for VO_2_ assessment [12]. Further investigation is needed in order to identify optimal techniques of metabolic assessment in ventilated patients.

It is important to acknowledge the limitations of this study. The small number of participants (n = 12) and the heterogeneity of these patients with regards to age and diagnosis means extrapolation of these results to all ICU patients is not justified. Technical issues resulted in incomplete data sets. Although the majority of the patients were deeply sedated two patients had a RASS score above − 2, The data from the motomed machine did not indicate if there was any active participation from the patient during the protocol. Although a possibility, such active participation would be expected to result in increases in oxygen uptake which was not seen.

## Conclusions

Measuring the metabolic demands of upper limb cycle ergometry in critically ill patients appears to be feasible and results in minimal increase in metabolic demands. Our study supports the notion that perceived benefit of early physical rehabilitation may outweigh concerns of adding to the physiological demands during the acute stages of illness. Further research is needed to determine how monitoring patient workload during rehabilitation could be used to personalise rehabilitation strategies during the course of their illness.

## Data Availability

The datasets generated during the current study are available from the corresponding author on reasonable request.

## Abbreviations

AKI: Acute kidney injury
BMI: Body mass index
BP: Blood pressure
CaO_2_: Arterial oxygen content
CO: Cardiac output
CO_2_: Carbon dioxide
CPAP: Continuous positive airway pressure
CvO_2_: Venous oxygen content
CVP: Central venous pressure
DO_2_: Oxygen delivery
ECG: Electrocardiogram
EMP: Early mobility programme
FeO_2_: Expired oxygen
FeCO_2_: Expired carbon dioxide
FiO_2_: Inspired oxygen
FiCO_2_: Inspired carbon dioxide
F/M: Female/ male
HR: Heart rate
Hb: Haemoglobin
ICU: Intensive care unit
IQR: Inter quartile ratio
LiDCO: Lithium dilution cardiac output
MV: Minute ventilation
N/ad: Noradrenaline
NOK/LR: Next of kin/living relative
O2: Oxygen
OD: Overdose
OOHA: Out of hospital arrest
PaO_2_: Partial pressure of oxygen
PEEP: Positive end expiratory peep
PiCCO: Pulse contour cardiac output
PS: Pressure support
RASS: Richmond agitation sedation score
REC: Research ethics committee
RR: Respiratory rate
SaO_2_: Blood oxygen saturation
ScvO_2_: Central venous oxygen saturation
SV: Stroke volume
VCO_2_: Carbon dioxide production
VO_2_: Oxygen uptake
VT: Tidal volume
